# Sodium Intensity Changes Differ Between Relaxation- and Density-Weighted MRI in Multiple Sclerosis

**DOI:** 10.3389/fneur.2021.693447

**Published:** 2021-07-14

**Authors:** Robert Stobbe, Annie Boyd, Penelope Smyth, Derek Emery, Diana Valdés Cabrera, Christian Beaulieu

**Affiliations:** ^1^Department of Biomedical Engineering, University of Alberta, Edmonton, AB, Canada; ^2^Department of Medicine, Division of Neurology, University of Alberta, Edmonton, AB, Canada; ^3^Department of Radiology and Diagnostic Imaging, University of Alberta, Edmonton, AB, Canada

**Keywords:** sodium, Na, multiple sclerosis, relaxation, MRI

## Abstract

**Introduction:** The source of Tissue Sodium Concentration (TSC) increase in Multiple Sclerosis (MS) remains unclear, and could be attributed to altered intracellular sodium concentration or tissue microstructure. This paper investigates sodium in MS using three new MRI sequences.

**Methods:** Three sodium scans were acquired at 4.7 T from 30 patients (11 relapsing-remitting, 10 secondary-progressive, 9 primary-progressive) and 9 healthy controls including: Density-Weighted (NaDW), with very short 30° excitation for more accurate TSC measurement; Projection Acquisition with Coherent MAgNetization (NaPACMAN), designed for enhanced relaxation-based contrast; and Soft Inversion Recovery FLuid Attenuation (NaSIRFLA), developed to reduce fluid space contribution. Signal was measured in both lesions (*n* = 397) and normal appearing white matter (NAWM) relative to controls in the splenium of corpus callosum and the anterior and posterior limbs of internal capsule. Correlations with clinical and cognitive evaluations were tested over all MS patients.

**Results:** Sodium intensity in MS lesions was elevated over control WM by a greater amount for NaPACMAN (75%) than NaDW (35%), the latter representing TSC. In contrast, NaSIRFLA exhibited lower intensity, but only for region specific analysis in the SCC (−7%). Sodium intensity in average MS NAWM was not significantly different than control WM for either of the three scans. NaSIRFLA in the average NAWM and specifically the posterior limb of internal capsules positively correlated with the Paced Auditory Serial Addition Test (PASAT).

**Discussion:** Lower NaSIRFLA signal in lesions and ~2× greater NaPACMAN signal elevation over control WM than NaDW can be explained with a demyelination model that also includes edema. A NAWM demyelination model that includes tissue atrophy suggests no signal change for NaSIRFLA, and only slightly greater NAWM signal than control WM for both NaDW and NaPACMAN, reflecting experimental results. Models were derived from previous total and myelin water fraction study in MS with T2-relaxometry, and for the first time include sodium within the myelin water space. Reduced auditory processing association with lower signal on NaSIRFLA cannot be explained by greater demyelination and its modeled impact on the three sodium MRI sequences. Alternative explanations include intra- or extracellular sodium concentration change. Relaxation-weighted sodium MRI in combination with sodium-density MRI may help elucidate microstructural and metabolic changes in MS.

## Introduction

Sodium MRI (NaMRI) has been applied to the study of multiple sclerosis (MS) for a decade and nearly all studies have focused on sodium density-weighted (NaDW) methods and tissue sodium concentration (TSC) measurement (1–11). However, the underlying microstructural or metabolic sources of the reported range of 20–80% greater TSC in lesions and 5–39% greater TSC in Normal Appearing White Matter (NAWM) remain unclear. These are generally thought to include increased intracellular sodium concentration and/or changes to the intra-/extracellular tissue space distribution (12), the latter also referred to as the cell volume fraction (13). Elevated intracellular sodium concentration may be associated with the proliferation of sodium channels and energetic failure as a result of demyelination (14); however, demyelination, axonal degeneration, hyper-cellularity, inflammation and edema may also all alter the cell volume fraction and sodium content. Sodium MRI sequences with differing contrast may help elucidate sources of TSC change, but have been limited in use to only two MS population studies as discussed below, both notably at 7T (15, 16).

Cell volume fraction calculation from NaDW images is based on constant intracellular (12 mM) and extracellular (145 mM) sodium concentrations (13), as well as the assumption that sodium within tissue exists in one or the other of these two spaces. However, a recent study measured excitation flip-angle dependent signal loss in human white matter which was attributed to residual quadrupole splitting (an effect of spin-3/2 nuclei expected in highly constrained and ordered environments) from ^23^Na nuclei within the tight sequential intra- and extracellular wraps of myelin water, constituting a distinct third environmental “space” for sodium MRI (17). TSC is then the sum of sodium within the stereotypical intra- and extracellular spaces as well as the myelin water space. Minimizing residual quadrupole signal loss (from the presumed myelin water space) is required to accurately measure complete TSC; however, standard 90° flip-angle excitation (used in almost every sodium MRI study) has been shown to exacerbate this loss, particularly in ordered white matter regions (17). Lower flip-angle NaDW imaging [as recommended in (17)] is used for the first time here in the context of MS to minimize residual quadrupole splitting and improve TSC measurement.

The first alternative sequence considered is an inversion recovery technique, which, when introduced, was suggested may weight signal toward the intracellular space (18). Others have also considered sodium inversion recovery in the context of MS measuring ~10% signal increase in acute lesions, a result attributed to either intracellular sodium accumulation or the hyper-cellularity of inflammation, as well as ~20% signal decrease in chronic lesions (15). Another sodium imaging technique labeled triple quantum filtering (TQF) has also been considered, and a lengthy 43 min TQF scan suggested global gray and white matter signal increase to be the result of intracellular sodium increase (presumably reflecting metabolic dysfunction) rather than simply an expansion of extracellular space due to demyelination and axon loss (16). The intracellular weighting natures of both inversion recovery and TQF imaging are based on differences in relaxation properties between intra- and extracellular space, T1 for inversion recovery and biexponential T2 for TQF. While recent opinion suggests that “intra- and extracellular sodium signals cannot be differentiated on the basis of relaxation time constant characteristics” (19), relaxation differences between these spaces in human brain remain unknown, may change with pathology, and may provide (possibly varying with pathology) *weighting* toward intracellular space. The inversion recovery technique considered here in MS for the first time is labeled sodium Soft Inversion Recovery FLuid Attenuation (NaSIRFLA) where the inclusion of a soft (i.e., longer) inversion RF pulse yields relaxation-weighting based on both T1 and biexponential T2 (18).

The second alternative sequence considered is labeled sodium Projection Acquisition with Coherent MAgNetization (NaPACMAN) (20). This technique is sensitive to macromolecular “ordered” tissue microstructure and has been shown to produce a “T2_fast_/T1-weighted” sodium image yielding greater contrast between white and gray matter compared to NaDW methods. It has not yet been applied to MS or any other neurological disorder.

Greater TSC values have been measured in advanced relapsing-remitting MS (RRMS) compared to early RRMS in lesions and both normal appearing white and gray matter (2). A few NaMRI studies on secondary and primary progressive MS (SPMS/PPMS) have also measured TSC increases in lesions and normal appearing white and gray matter that are greater in SPMS than in RRMS (11), and in both SPMS and PPMS than RRMS (3), but with different cerebral distributions of higher sodium between SPMS and PPMS as assessed by voxel-based image analysis (4). However, only one NaMRI study has included all three MS subtypes (3).

Here, in addition to a modified NaDW sequence for improved TSC accuracy, two alternative sodium MRI sequences with contrast beyond TSC were explored for the additional information they may provide in the context of relapsing-remitting, secondary-progressive, and primary progressive multiple sclerosis (RRMS, SPMS, PPMS). The purposes of this study were to: (i) investigate sodium signal differences in lesions across the entire brain and specific regions of NAWM for three complementary sodium MRI methods at 4.7 T (NaDW, NaSIRFLA, and NaPACMAN), (ii) determine whether the various sodium signal changes are similar across three subtypes of MS (RRMS, SPMS, and PPMS), and (iii) examine correlations of regional cerebral sodium signal with cognitive and clinical scores.

## Methods

### Participants

Written informed consent was obtained for all 39 participants, and the study was approved by the Human Research Ethics Board committee of the University of Alberta. Participants recruited for the study included 9 healthy controls with no prior diagnosed neurological disorders, as well as 30 MS participants: 11 relapsing-remitting (RRMS), 10 secondary progressive (SPMS), and 9 primary progressive (PPMS). A more detailed description of the participant cohort can be viewed in Table 1. All MS patients had a confirmed diagnosis by a clinician based on the revised McDonald criteria (21) and were referred to our research team if they had interest in participating in our clinical research study. At the time of the MRI, none of the participants were experiencing a relapse nor using steroid therapies, but information regarding previous relapses was not available. Of the 11 RRMS participants, 3 were not undergoing treatment, but 8 were on various disease-modifying therapies (dimethyl fumarate, glatiramer acetate, fingolimod, and natalizumab). Of the SPMS and PPMS participants, none were receiving treatment with the exception of one SPMS participant on IV infused natalizumab. There is a large overlap in the participant cohort with a previously published DTI study of limbic tracts by Valdes Cabrera et al. (22).

**Table 1:** Participant demographics expressed as mean ± SD with (min-max) listed below.

	**Controls**	**RRMS**	**SPMS**	**PPMS**	**MS All**
*N*	9	11	10	9	30
Sex (M:F)	3:6	2:9	3:7	6:3	11:19
Age (years)	50 ± 16 (27–75)	40 ± 12^a^ (21–58)	55 ± 7 (45–66)	54 ± 7 (41–65)	49 ± 11 (21–66)
EDSS	-	3.5 ± 1.5^b^ (1.5–6.0)	5.4 ± 1.7 (3.0–8.5)	5.8 ± 1.0 (4.0–7.0)	4.8 ± 1.7 (1.5–8.5)
Time since diagnosis (years)	-	9 ± 9 (1–28)	23 ± 10^c^ (2–34)	7 ± 6 (3–17)	13 ± 11 (1–34)
TLV (*cm*^3^)	-	3.1 ± 3.1 (0.23–8.8)	8.8 ± 8.8 (0.19–27.9)	10.1 ± 11.2 (0.83–37.2)	7.1 ± 8.5 (0.19–37.2)
MSIS-29	-	95 ± 42 (38–168)	75 ± 16 (38–97)	89 ± 14 (73–112)	86 ± 28 (38–168)
PASAT	-	44 ± 7 (37–54)	43 ± 12 (19–59)	37 ± 7 (30–49)	42 ± 9 (19–59)
SDMT	-	52 ± 9 (36–71)	57 ± 16 (34–89)	47 ± 13 (23–62)	52 ± 13 (23–89)
BVMT-R	-	23 ± 8 (10–34)	22 ± 9 (11–34)	18 ± 10 (5–30)	21 ± 9 (5–34)
T25W (s)	-	6.5 ± 2.3 (3.6–12.0)	10.2 ± 5.8 (5.3–19.5)	10.0 ± 5.6 (5.8–20.6)	8.5 ± 4.7 (3.6–20.6)
9-HPT dominant (s)	-	24.3 ± 6.0 (19.1–40.0)	26.6 ± 6.6 (18.5–40.9)	32.2 ± 10.3 (20.5–55.0)	27.5 ± 8.2 (18.5–55.0)
9-HPT Non-dominant (s)	-	25.4 ± 5.7 (18.2–39.6)	30.3 ± 14.6 (20.0–67.3)	38.4 ± 17.2 (21.0–75.4)	31.0 ± 13.7 (18.2–75.4)

a *The RRMS subgroup was significantly younger than both the SPMS and PPMS subgroups.*

b *The RRMS subgroup had EDSS scores significantly lower than both the SPMS and PPMS subgroups.*

c *The SPMS subgroup had been living with an MS diagnosis significantly longer than both RRMS and PPMS subgroups*.

^*^EDSS score was not reported for two PPMS participants, time since diagnosis was unknown for one SPMS participant, two SPMS and one PPMS participants had not completed the PASAT, one SPMS participant was unable to complete the BVMT-R, two SPMS and one PPMS participant were unable to complete the T25W, and one SPMS participant was unable to complete both the dominant and non-dominant 9-HPT.

### Functional Testing

Trained MS nurses administered all cognitive and clinical tests which were used to describe the demographics of the study, and to correlate with NaMRI. The evaluation consisted of: the Multiple Sclerosis Impact Scale (MSIS-29) for the impact of MS from the patient's perspective, Paced Auditory Serial Addition Test (PASAT) for auditory information processing speed and flexibility, Symbol Digit Modalities Test (SDMT) for cerebral dysfunction and slowed processing of information, Brief Visuospatial Memory Test-Revised (BVMT-R) for visuospatial memory (total recall score only), both the dominant and non-dominant 9-Hole Peg Test (9HPT) for upper extremity function, and Timed 25-Foot Walk (T25W) for lower extremity function. Kurtzke Expanded Disability Status Scale (EDSS) scores, which characterize general disability, and time since diagnosis were obtained from clinical reports.

### MRI Acquisition

MRI acquisition was performed using a Varian Inova 4.7 T MRI with broadband capabilities. The two 1H scans included: Fast Spin-Echo Fluid-Attenuated Inversion Recovery (FSE FLAIR) with FOV 256 × 192 *mm*^2^, 1 × 1 *mm*^2^in-plane resolution, 38 4-mm slices (no gap), TR = 34 s, 32 variable flip-angle echoes with echo-spacing of 8.5 ms, TE = 204 ms, TI = 3,000 ms, and scan time 3:24 min; Diffusion Tensor Imaging (DTI) using single-shot spin-echo Echo-Planar Imaging, GRAPPA R = 2, 80 1.7 mm slice (no gap) with coverage of 136 mm, FOV 218 × 238 *mm*^2^, matrix 128 × 140, 1.7 × 1.7 × 1.7 = 4.9 *mm*^3^ voxel resolution, zero-filled to 0.85 × 0.85 *mm*^2^ in-plane, TR = 9,500 ms, TE = 54 ms, 30 directions, *b* = 1,000 s/*mm*^2^, 5 *b*_0_ and scan time 6:13 min. While DTI analysis formed a separate study (22), here DTI images were used to identify specific white matter regions.

Three different NaMRI scans were acquired using a home-built single-tuned birdcage RF coil: (i) Sodium Density-Weighted (NaDW) using Twisted Projection Imaging (TPI), flip angle 30, RF pulse duration (τ_RF_) = 0.11 ms, TR = 85 ms, TE = 0.11 ms, 3.2 × 3.2 × 6.4 *mm*^3^ voxel resolution and scan time of 8.5 min; (ii) Sodium Projection Acquisition with Coherent MAgNetization (NaPACMAN) using TPI, flip angle 110, τ_RF_ = 4.0 ms, TR = 25 ms, TE = 2.5 ms, 3.2 × 3.2 × 6.4 *mm*^3^ voxel resolution, and scan time of 7.5 min (20); and (iii) Sodium Soft Inversion Recovery FLuid Attenuated (NaSIRFLA) using TPI, flip angle 64, τ_RF_ = 0.32 ms, soft inversion RF = 180 for 5.0 ms, TR = 150 ms, TI = 37 ms, TE = 0.22 ms, 4.5 × 4.5 × 9.0 *mm*^3^ voxel resolution, and scan time of 7:50 min (18). Voxel resolution is defined by $\frac{1}{\left(2k_{\max}\right)},$ and for each scan voxels are stretched along the inferior-superior dimension. This was done for time/SNR efficiency and to “match” the anisotropic nature of the FLAIR voxels (1 × 1 × 4 *mm*^3^). The NaDW scan included a smaller flip angle (30°) than the typical 90°. This smaller flip angle with short τ_RF_ was used to mitigate signal loss associated with residual quadrupole splitting (17). Note that with smaller flip angle, TR can also be reduced without introducing T1-weighting. Phantom tubes (diameter = 3 cm, length = 11 cm) with 5% agar and known sodium concentration of 64 mM were placed in the scanner bilaterally adjacent to the head for reference. All measured image intensities are given relative to the calibration tube values. This was done to facilitate equivalent data representation for the three sequences, as NaPACMAN and NaSIRFLA signals cannot be converted into TSC values. As described in the Results, TSC values for NaDW can calculated by multiplying the relative image intensities listed by 64 mM. This yields sodium values in terms of mmol per 1L of tissue (written as mmol/L-tissue), acknowledging that tissue is not a simple “solution.” There was no NaPACMAN for one PPMS participant and no NaSIRFLA for one RRMS participant.

### Image Analysis and Testing

Lesions were identified as local regions of hyperintensity on FLAIR images, and 397 regions (volumes) of interest (ROI) over all 30 MS participants were hand-drawn on these images. All three sodium scans were then coregistered to FLAIR (SPM8) to yield average image intensity within the lesions. For specific white matter tract analysis, the FLAIR and *b*_0_ images of the DTI data were coregistered to the sodium images using FSL FLIRT with 12 degrees of freedom. These transformations were then applied to the primary eigenvector direction color-coded fractional anisotropy (FA) maps derived from DTI images processed following the protocol outlined in a previous publication by us (22). The primary eigenvector color FA maps were used as a guide to outline manual ROIs for large, easily segmented white matter tracts including: the splenium of corpus callosum (SCC), the anterior limb of internal capsule (ALIC) and the posterior limb of internal capsule (PLIC). The SCC was defined as the posterior ¼ of the corpus callosum when viewed on a midsagittal slice. The ALIC was traced from the internal capsule genu (ICg) to the external capsule (EC) anteriorly from a level superior to the anterior commissure until the IC was no longer distinguishable from the EC. The PLIC was traced from the ICg to the EC posteriorly, from a level where the PLIC was clearly identifiable, superiorly to where the IC is no longer distinguishable from the EC. All structures were outlined on multiple slices and a mean of the sodium signal intensity from voxels across all slices was calculated per scan type. Control WM and average NAWM sodium intensity is the average intensity in the three WM regions considered. One healthy control and one PPMS participant had no color-coded FA maps and regional ROIs were directly drawn on the FLAIR using identifiable landmarks.

FLAIR images were used to create ventricular and longitudinal fissure CSF masks (based on regions with no signal) which were convolved with the sodium point-spread-function (PSF) of saline (${\text{T}}_{2}^{*}$ = 53 ms at 4.7T). The CSF masks were then expanded to all voxels in which the PSF convolution yielded values >0.025 (or 2.5% of full value). At 2.5%, the CSF contributes only an effective 3.75 mmol/L-tissue worth of signal to voxel (assuming CSF = 150 mM). A CSF contribution greater than this was considered too large for tissues of interest. The expanded CSF masks were used to eliminate CSF contaminated voxels from the white matter ROIs. Note that other white matter regions such as the body and genu of corpus callosum did not retain sufficient voxels uncontaminated by CSF across all patients. This is the reason for the focus on the SCC, PLIC and ALIC. White matter ROIs were further segmented into regions containing NAWM and lesions as identified on coregistered FLAIR.

NAWM and lesion sodium intensity for the different scan types were compared with healthy controls and between the different MS subtypes using unpaired *t*-tests at a false discovery rate (FDR) for multiple comparisons adjusted significance threshold of *q* < 0.05. Both healthy control WM and NAWM were tested for correlation with age using standard linear regression (FDR *q* < 0.05). Paired *t*-tests were used for intra-patient comparison of lesions with NAWM in the same WM region. Intra-patient sodium lesion intensity relative to directly comparable NAWM was then tested for correlation between the scan types to determine whether lesions with greater TSC (NaDW) increase are also associated with greater increases or decreases on NaPACMAN and NaSIRFLA (FDR *q* < 0.05). Similarly, NAWM intensity relative to control WM was also tested for correlation between the sequences. NAWM sodium intensity from the three different scan types across MS participants was tested for correlation (Partial Pearson, accounting for age and sex) with disease duration, EDSS, MSIS-29, PASAT, BVMT-R, SDMT, T25W, 9HPT dominant, and 9HPT non-dominant scores. Total lesion volume (TLV) was also tested for correlation with clinical/cognitive tests. A relatively large FDR adjusted threshold of *q* < 0.2 was used in this investigatory study of only 30 MS patients to avoid discarding potentially significant sodium MRI correlations with clinical/cognitive tests.

## Results

### Cohort Description

A full description of the cohort demographics can be found in Table 1. There were almost double the number of females in the study (14M:25F). For the healthy controls, RRMS, and SPMS subgroups, the females outnumbered the males (3:6, 2:9, 3:7 M:F, respectively), but in the PPMS subgroup there were more males than females (6M:3F). The age ranged from 21 to 75 years for the entire cohort with a mean age of 50 ± 16 years for the healthy controls (*n* = 9) and 49 ± 11 years for all MS participants (*n* = 30). The RRMS subgroup was significantly younger (40 ± 12 years) than both the SPMS (55 ± 7 years, *p* = 0.019 and PPMS (54 ± 7 years, *p* = 0.032) subgroups. The EDSS score ranged from 1.5 to 8.5 and the RRMS subgroup had significantly lower EDSS score (3.5 ± 1.5) than both progressive subtypes (SPMS 5.4 ± 1.7, *p* = 0.019; PPMS 5.8 ± 1.0, *p* = 0.010). Note that these numbers are simply average values from the individually discrete (in 0.5 increments) EDSS scores. Furthermore, the SPMS group had been living with an MS diagnosis for a significantly longer time (23 ± 10 years) than both RRMS and PPMS subtypes (RRMS 9 ± 9 years, *p* = 0.003; PPMS 7 ± 6 years, *p* = 0.002).

### Average NAWM and Lesion Analysis for All MS Patients

**Figure 1:**
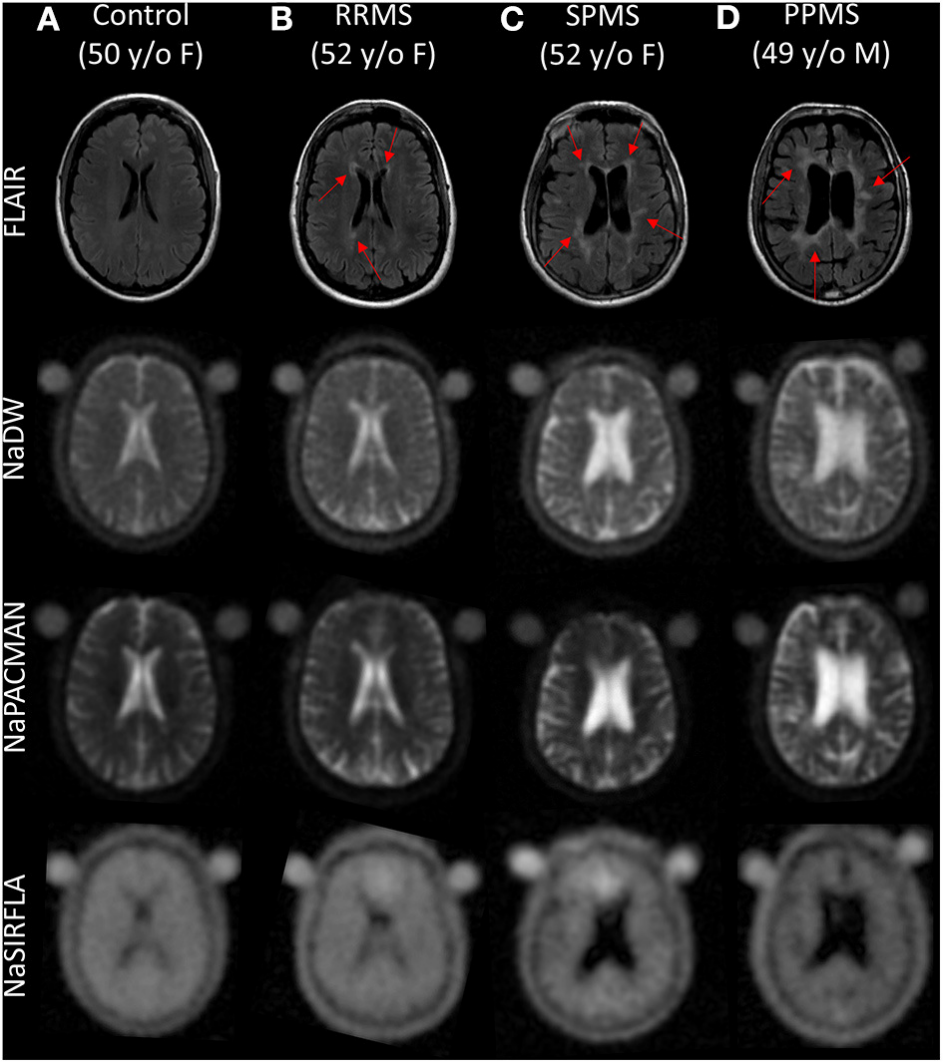
Qualitative axial image comparison of **(A)** a healthy control and **(B–D)** three MS subtypes for FLAIR, NaDW, NaPACMAN, and NaSIRFLA. Regions of hyperintensity on FLAIR (red arrows) have visibly greater sodium signal compared to NAWM on NaDW and NaPACMAN (hyperintense), where NaPACMAN shows the greatest lesion contrast. Lesion sodium intensity on NaSIRFLA is difficult to visually distinguish from NAWM, but is slightly hypointense. Note that NaSIRFLA images display an artifact of hyperintensity in the frontal sinus cavity region due to off-resonance effects.

**Figure 2:**
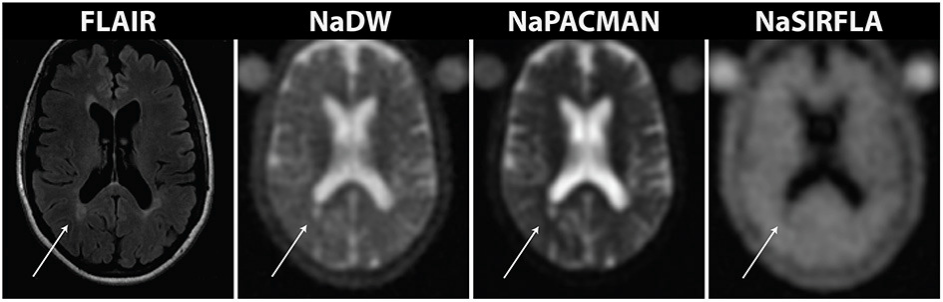
Images from a 58 year old female RRMS patient highlight image intensity differences between the three sodium MRI sequences. The lesion identified with the arrow exhibits greater signal enhancement on NaPACMAN than NaDW, while the same lesion has lower image intensity on NaSIRFLA.

Representative sodium images from a healthy control and each of the three MS sub-types are shown in Figure 1. Regions of hyperintensity identified on FLAIR are visibly increased on both NaDW and NaPACMAN. This is particularly apparent when one considers large lesions. Signal difference between lesions and NAWM is less apparent for NaSIRFLA, however slight hypointensities are present. Lesion hypointensity on NaSIRFLA is visibly apparent in Figure 2, where images of an RRMS patient also show clearly greater lesion intensity increase for NaPACMAN compared to NaDW.

**Figure 3:**
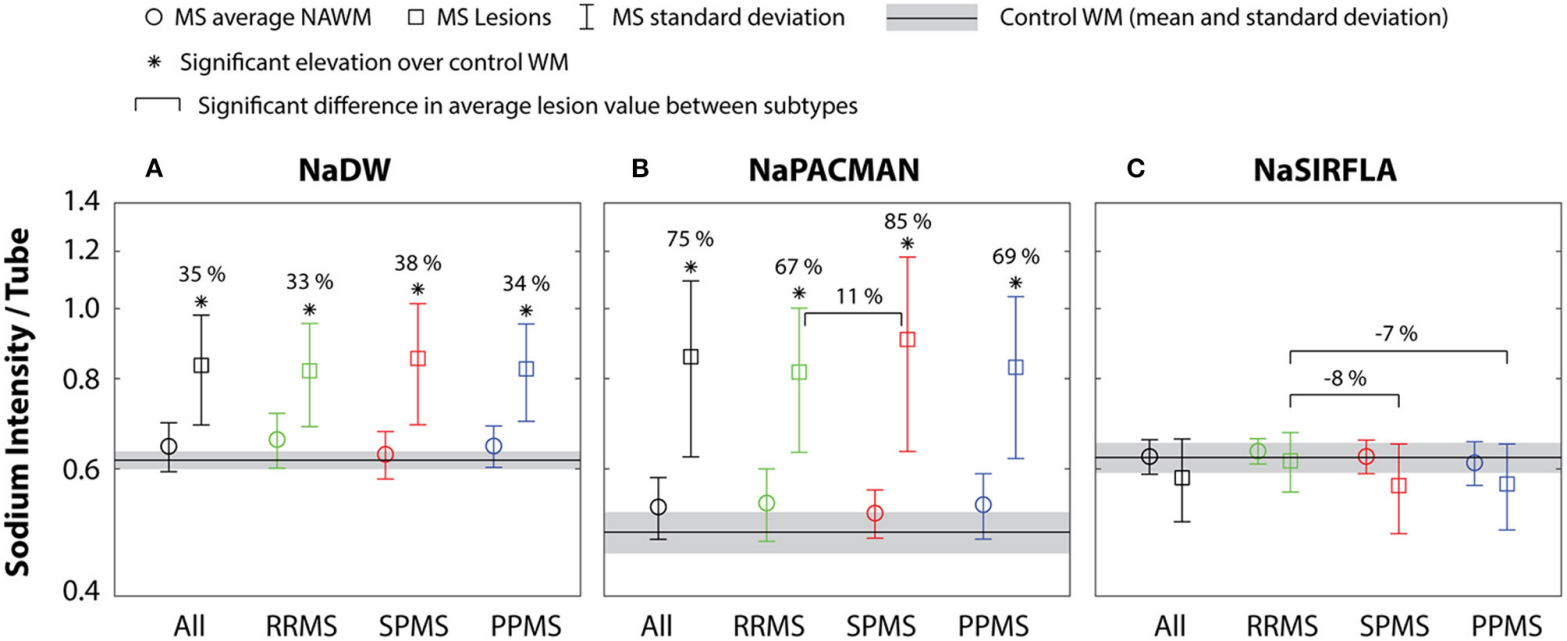
**(A)** NaDW and **(B)** NaPACMAN both yield significantly greater sodium intensity in lesions (squares) than control WM for all MS subtypes, but this increase is ~2× greater for NaPACMAN than NaDW. NaPACMAN shows a significantly higher lesion intensity in the SPMS subgroup relative to the RRMS subgroup. **(C)** Lesions do not differ from control WM for NaSIRFLA; however, the SPMS and PPMS subgroups exhibited lower signal than the RRMS subgroup. No significant differences between MS NAWM (circles) and control WM were measured for any of the three sequences.

Control WM yielded sodium intensity values relative to the agar tubes of (mean ± standard deviation): NaDW 0.62 ± 0.02 (or TSC = 40 ± 1 mmol/L-tissue), NaPACMAN 0.49 ± 0.03, and NaSIRFLA 0.62 ± 0.03 (Figure 3). The average relative NAWM sodium intensity over all MS patients was 0.64 ± 0.05 (or TSC = 41 ± 3 mmol/L-tissue) for NaDW (Figure 3A), 0.53 ± 0.05 for NaPACMAN (Figure 3B) and 0.62 ± 0.03 for NaSIRFLA (Figure 3C); however, these values were not significantly different than control WM. Neither MS NAWM nor control WM significantly correlated with age for any of the sequence types. The 397 lesions over all MS patients yielded average sodium intensities of 0.83 ± 0.14 for NaDW (or TSC = 53 ± 9 mmol/L-tissue) (Figure 3A), 0.86 ± 0.23 for NaPACMAN (Figure 3B), and 0.57 ± 0.08 for NaSIRFLA (Figure 3C). Average lesion intensity was 35 % (*p* < 0.001) greater than control WM for NaDW and 75% (*p* < 0.001) greater for NaPACMAN.

### Average NAWM and Lesion Analysis Between MS Subtypes

There was no significant difference in relative sodium intensity on NaDW (and thus measured TSC) between the three MS subtypes for either lesions or NAWM (Figure 3A). However, the mean lesion intensity of SPMS patients on NaPACMAN was 11 % (*p* = 0.002) greater than RRMS patients (Figure 3B). For NaSIRFLA, the mean lesion intensities of SPMS and PPMS patients were −8% (*p* < 0.001) and −7% (*p* < 0.001) less, respectively, than RRMS patients (Figure 3C).

### Region Specific NAWM and Lesion Analysis

**Figure 4:**
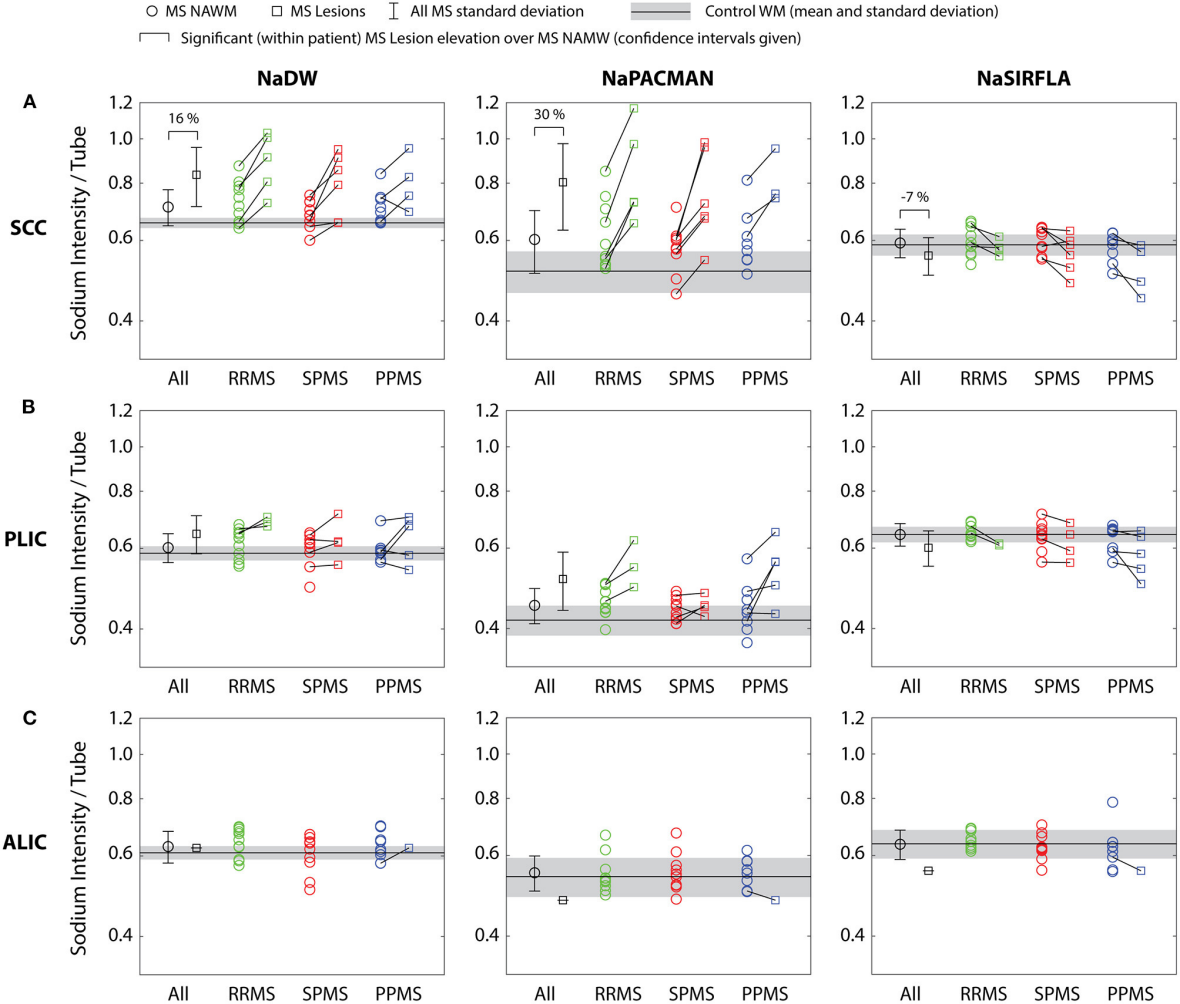
**(A)** Lesions within the SCC have significantly greater signal than adjacent, directly comparable NAWM for both NaDW and NaPACMAN, with NaPACMAN yielding a relative difference ~2× greater than NaDW. Alternatively, NaSIRFLA shows a significant signal decrease in SCC lesions. **(B,C)** There were no significant findings in the PLIC or ALIC.

Direct comparison of lesions and NAWM within the same WM structure yields significantly greater lesion intensity in the SCC for both NaDW at 16% (*p* < 0.001) and NaPACMAN at 30% (*p* < 0.001). However, the relative difference between lesions and NAWM is ~2× greater for NaPACMAN. For NaSIRFLA, lesion intensity is 7% (*p* < 0.001) less than NAWM in the SCC (Figure 4). NAWM image intensity was not significantly different than control WM for any of the three white matter sub-regions.

### Lesion Intensity Correlation Between Sequences

**Figure 5:**
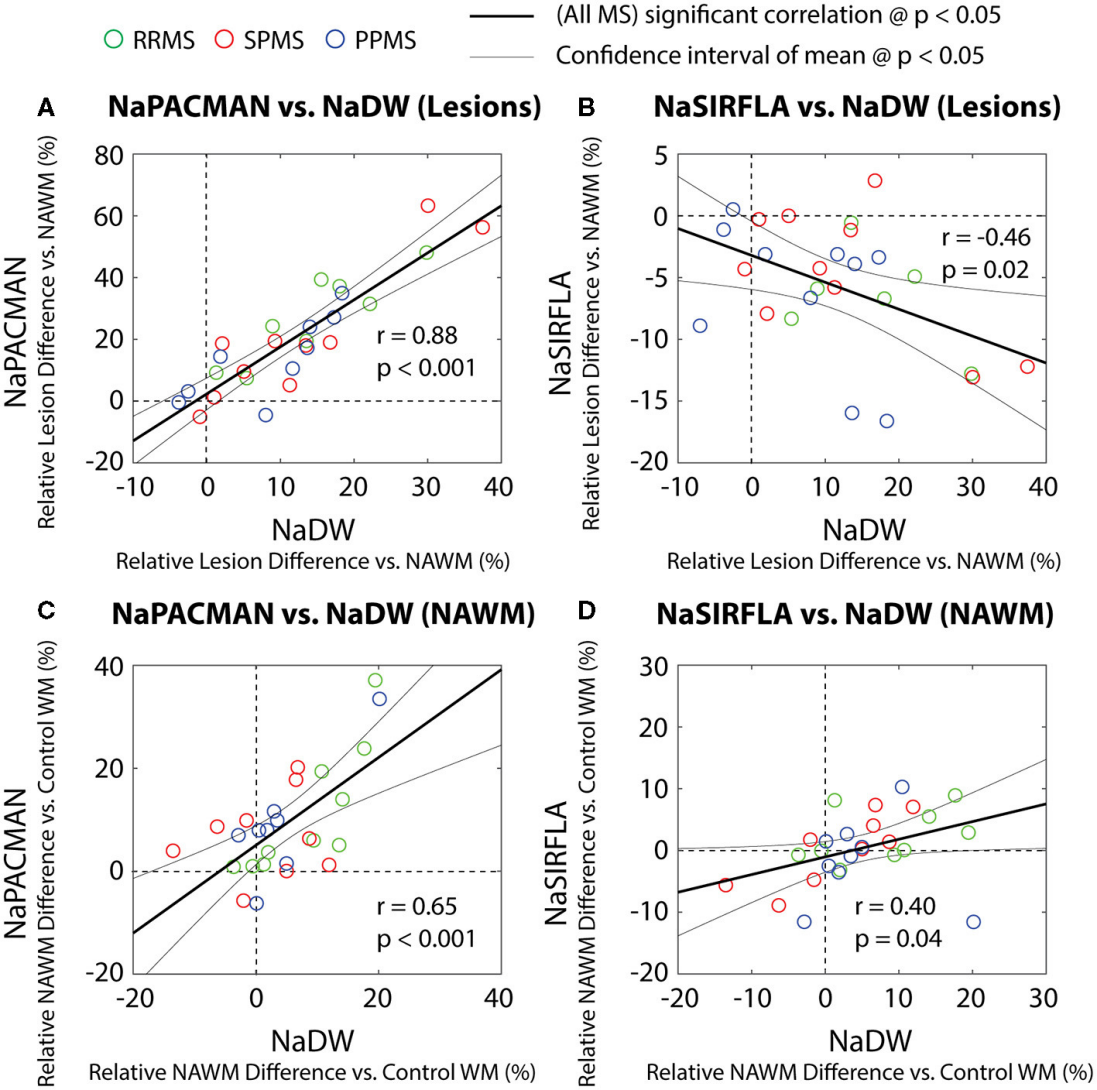
Relaxation-weighted sequence correlations with NaDW (or TSC) for relative lesion intensity difference from directly comparably NAWM (in the same MS patient and WM region) **(A,B)** and relative NAWM difference from average control WM **(C,D)** for all 30 MS participants (one symbol per patient). **(A)** Lesion intensity increase over directly comparable NAWM is positively correlated between NaPACMAN and NaDW with slope = 1.52 ± 0.33 showing a greater change for NaPACMAN. **(B)** However, lesion vs. NAWM is negatively correlated between NaSIRFLA and NaDW with slope = −0.22 ± 0.18 (i.e., the more sodium TSC in lesions, the greater the signal loss on NaSIRFLA) and significant intercept offset of −0.03 ± 0.03 (*p* = 0.02). Note that for **(A)** the y-axis is 2× the x-axis, while in **(B)** the y-axis is 0.5× the x-axis. **(C)** NAWM intensity relative to average control WM is positively correlated between NaPACMAN and NaDW with slope = 0.85 ± 0.41 (less steep than lesions vs. NAWM) and significant intercept offset of 0.05 ± 0.04 (*p* = 0.01). **(D)** NAWM vs. control WM is positively correlated between NaSIRFLA and NaDW with slope = 0.29 ± 0.27; this is opposite that seen for lesions relative to NAWM.

Over all 30 MS patients, the relative lesion intensity difference from directly comparable NAWM (the same MS patient and WM region) was correlated between NaDW and NaPACMAN (*r* = 0.88, *p* < 0.001) showing that PACMAN had ~1.5× the signal changes in lesions compared to NaDW (Figure 5A). Relative lesion intensity difference from directly comparable NAWM was negatively correlated with NaDW for NaSIRFLA (*r* = −0.46, *p* = 0.02) (Figure 5B). However, the relative NAWM intensity difference from average control WM was positively correlated with NaDW (or TSC) on both NaPACMAN (*r* = 0.65, *p* < 0.001, Figure 5C) and NaSIRFLA (*r* = 0.40, *p* = 0.04, Figure 5D).

### MS NAWM Correlation With Functional Testing

**Figure 6:**
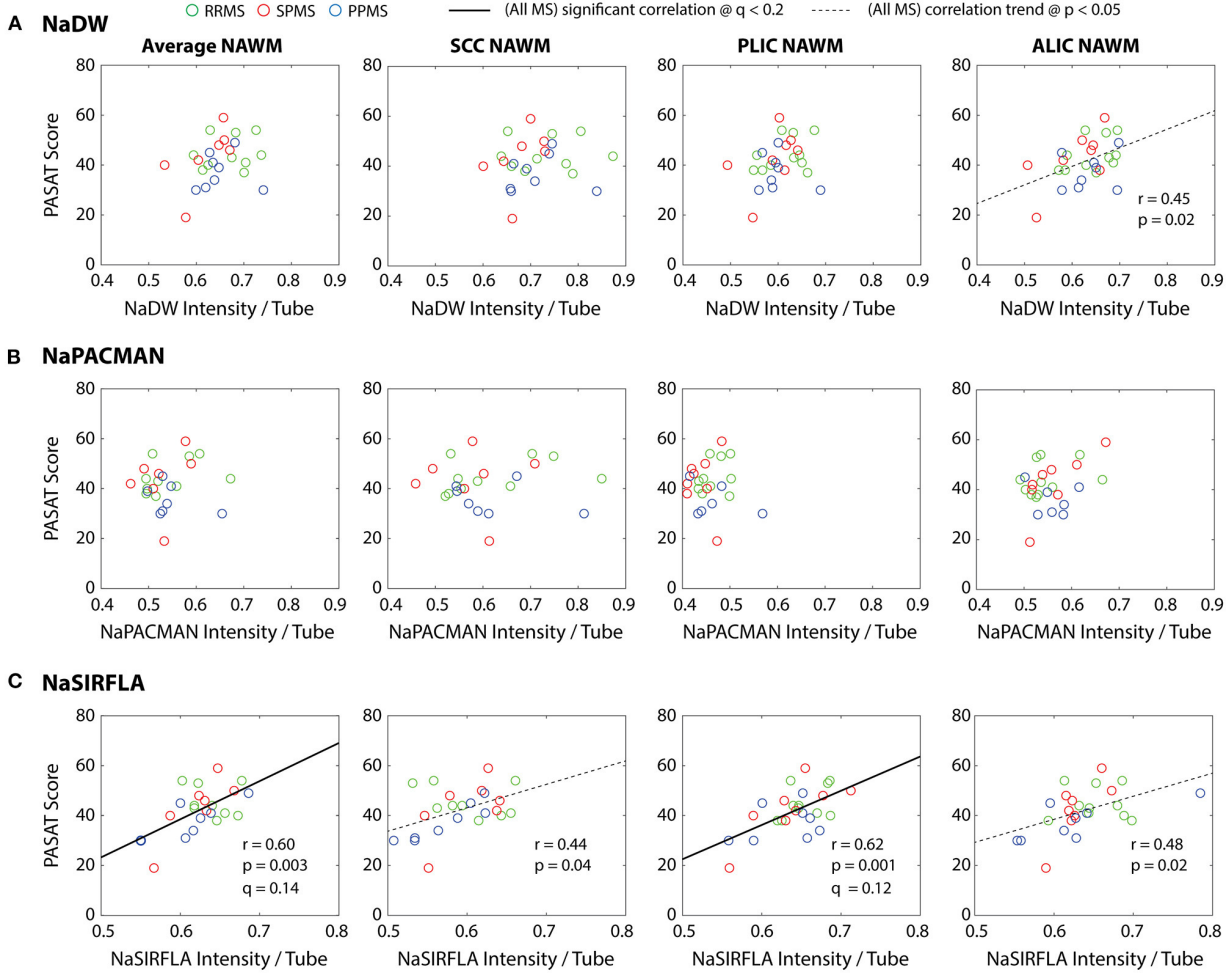
**(C)** NaSIRFLA in the PLIC NAWM and across the average of all three regions considered significantly correlates positively with PASAT. Similar positive NaSIRFLA trends are observed in both the SCC and ALIC NAWM that do not survive false discovery rate correction. **(A)** A positive NaDW trend that does not survive false discovery rate correction is also observed in the ALIC NAWM. **(B)** No correlations or trends were observed for NaPACMAN. Age and sex have little effect on the partial correlation, and thus for display purposes the regression lines shown are those of direct correlation. Note that Na intensity on these three scans did not correlate with the other cognitive and clinical scores (data not shown).

NaSIRFLA intensity positively correlated with raw PASAT score in the PLIC NAWM (*q* = 0.08) and the average NAWM over all three WM regions (*q* = 0.11) (Figure 6). No other cognitive or clinical tests yielded significant correlations with sodium intensity on the three scans that survived the false discovery rate threshold of *q* < 0.2.

## Discussion

### Sodium MRI in NAWM

Previously published papers have measured 5–39% greater TSC in the NAWM of MS patients compared to control WM: e.g., 39% (1), 9% (3, 11), 7% (5), and 5% (2). Here, average TSC (reflected by low flip angle NaDW) across the NAWM of all MS patients was not significantly greater than control WM. Each of the previous studies used center-out ^23^Na MRI acquisition techniques with short TE to minimize ${\text{T}}_{2}^{*}$ related signal loss; however, they all also used large flip-angle (80–90°) RF excitation pulses. Large flip-angle excitation has been shown to yield signal loss in healthy brain WM, a result attributed to residual quadrupole splitting (an effect related to the spin-3/2 nature of ^23^Na) in dense and highly ordered spaces as may be found within the wraps of myelin, thus underestimating TSC in healthy brain WM (17). Loss of myelin may result not only in actual TSC increase (assuming macromolecule replacement by fluid) but also replacement of the “less visible” nuclei within the myelin wraps (or weighted lower signal) with “completely visible” nuclei, yielding apparently greater relative TSC increase than the actual case. Very short RF pulses (τ_RF_ = 0.11 ms) with small flip angles (30° in this study) minimize residual quadrupole signal loss, yielding signal change more accurately reflecting TSC change (17), which in the case of NAWM in MS is a small change. Measurements of much greater TSC in NAWM than healthy controls [e.g., 39% in (1)] may reflect differences in image quality and analysis, particularly pertaining to partial volume averaging with CSF due to point spread function (PSF) smearing and increased brain atrophy with more CSF in MS patients.

There were no significant NAWM differences between the MS subgroups, but the subgroup sample sizes are small. In contrast, a previous study including all MS subtypes measured significantly greater TSC in the NAWM of SPMS and PPMS patients compared to healthy controls, but not RRMS patients (3), and significantly greater TSC in SPMS compared to RRMS patients. Another more recent study also measured a trend for greater TSC in NAWM of SPMS compared to RRMS patients (11). While the previous studies included ~100 total subjects to our 39 total subjects (MS and controls), differences in measured NAWM values between subgroups may also reflect different analyses. CSF contaminated voxels were rigorously excluded from the NAWM regions analyzed in this study. This was the reason for the focus on the PLIC and ALIC which do not border CSF, as well as the focus on the large SCC region. Increased ventricle (or other CSF) volumes, as may be seen in older progressive MS patients, can result in greater TSC (or apparent TSC) bias to atrophy in more distant voxels than those simply adjacent to CSF space as a result of PSF smearing. NAWM in multiple sclerosis may also be affected to varying extents in different white matter regions. Others have also identified the SCC as a NAWM region of particularly elevated TSC in progressive MS patients, with no significant increase in the PLIC or ALIC (4).

NaSIRFLA intensity in NAWM is not significantly different than control WM for the MS population or specific MS subgroups. While other papers have promoted the intracellular weighting of NaSIRFLA (18, 23, 24), the result measured here does not exclude the possibility that the intracellular sodium concentration in NAWM of MS patients is different than that of controls. For example, a smaller intracellular volume fraction in NAWM of MS patients may reduce the total received signal from the intracellular weighted NaSIRFLA sequence, offsetting any intracellular sodium concentration increase. Another paper using a purportedly selectively intracellular ^23^Na imaging technique labeled triple quantum filtering in combination with TSC MRI calculated (from their image data) both significant intracellular sodium concentration increase and significant intracellular volume fraction decrease in the NAWM of RRMS patients (16). It is possible that this may also explain the NaSIRFLA result of Figure 3. However, accurate calculation of intracellular ^23^Na concentration with any imaging technique deriving signal weighting characteristics from differences in relaxation between compartments (i.e., both NaSIRFLA and triple quantum filtering) requires knowledge of both intra- and extracellular relaxation and the extent of difference between them. These two relaxation values have not been measured in human brain.

### Sodium MRI in FLAIR-Identified Lesions

The mean TSC measured in all 397 lesions over all MS patients was 53 ± 9 mmol/L-tissue or 35 ± 9% greater than average control WM (Figure 3A). This value is in the middle of the range of mean TSC proportional elevations reported in previously published papers: 17–82% (1–5, 8, 11). However, sodium MRI lesion intensity has previously been shown to vary with MS lesion size as a result of PSF smearing related to rapid ${\text{T}}_{2}^{*}$ decay, where PSF smearing results in large sodium underestimation for small MS lesions (25). Identified lesions included had an average lesion volume of 0.22 mL (or 3.3 voxels, minimum ~1 voxel). In our previous study of lesions in PPMS patients (a subset of the patients in this study) we suggested that even lesions as large as 8 voxels may be under-estimated by as much as 40% to 80% on account of PSF smearing, and that minimum lesion volumes of at least 50 voxels (or 3.2 mL in this case) may be required to reduce this under-estimation to <20% for the imaging scenario of this study. Given the PSF related underestimation of sodium in lesions and the image intensity dependence on lesion volume, no attempt was made to correlate sodium MRI lesion intensity with functional test or clinical score [although others have shown correlations between lesion TSC and clinical score (1, 3, 11)]. Lesions were primarily included in this analysis for intensity comparison between sodium sequence types (as discussed below). Note that lesion intensity dependence on lesion size can explain the large standard deviation in the lesion TSC measurements of this study (and potentially across studies). PSF smearing can also explain the significantly greater image intensity in lesions of SPMS patients on NaPACMAN and the significantly less image intensity in lesions of SPMS and PPMS patients on NaSIRFLA. Both progressive MS subgroups contain (on average) larger total lesion volume than the RRMS patients (Table 1). Note that different TSC values have also been measured in different lesion types (Gd-enhancing > T_1_-hypointense > T_1_-isointense > lesion with reduced diffusion) (5), but in the current paper T_1_ images were not acquired due to time constraint and thus different lesion types could not be identified. Finally, a longitudinal study in RRMS patients showed that TSC elevations in lesions disappeared with regression of vasogenic edema and Gd enhancement (6). Our study was cross-sectional and did not use Gd to detect active lesions.

The average lesion sodium signal was 35% greater than control WM for NaDW (Figure 3A), but 75 % greater for NaPACMAN (Figure 3B). Although these values represent mean lesion intensity over all patients and lesions with respect to mean control WM values, and do not take into account varying lesion and control values with WM regions (Figure 4), direct comparison of lesions with corresponding NAWM in the same MS patient also demonstrates significantly greater lesion intensity (over NAWM) for NaPACMAN than NaDW, 1.5 times greater in this case (Figure 5A), as well as NaSIRFLA lesion signal decrease that correlated with NaDW at a rate of −0.22 (Figure 5B). Significantly greater lesion signal for NaPACMAN than NaDW and significantly less lesion signal for NaSIRFLA argues that (average) tissue sodium relaxation must be substantially altered in FLAIR-identified lesions. A more fluid-like “average lesion microenvironment” could explain the contrast of both NaPACMAN, which is weighted toward less macromolecularly dense and ordered microenvironments, and NaSIRFLA, which is inherently a fluid suppression technique.

### Sodium Image Contrast Modeling—The Inclusion of Sodium in Myelin

Previous sodium MRI studies have only assumed two sodium compartmental spaces (intra- and extracellular space) to arrive at either intracellular sodium concentration estimates (24), or measures of cell volume fraction (CVF) (13, 24, 26). However, recent work suggests that myelin water may constitute a third tissue “compartment” with distinctly different ^23^Na MRI relaxation characteristics than either intra- or extracellular space (17). Because myelin is composed of very thin (30 Å) sequential wraps of intra and extracellular space (27), it creates a highly constrained and ordered environment, and while a short T_2_ component is attributed to myelin water for ^1^H MRI (27), residual quadrupole splitting has been attributed to myelin sodium for ^23^Na MRI (17). According to Laule et al. (27), the thicknesses of both the intra- and extracellular spaces in myelin are the same, and thus the average myelin water sodium concentration is a simple average of the intra- and extracellular [Na+] values of 12.5 and 145 mM, respectively [middle range values from (28)], or 78.75 mM.

**Figure 7:**
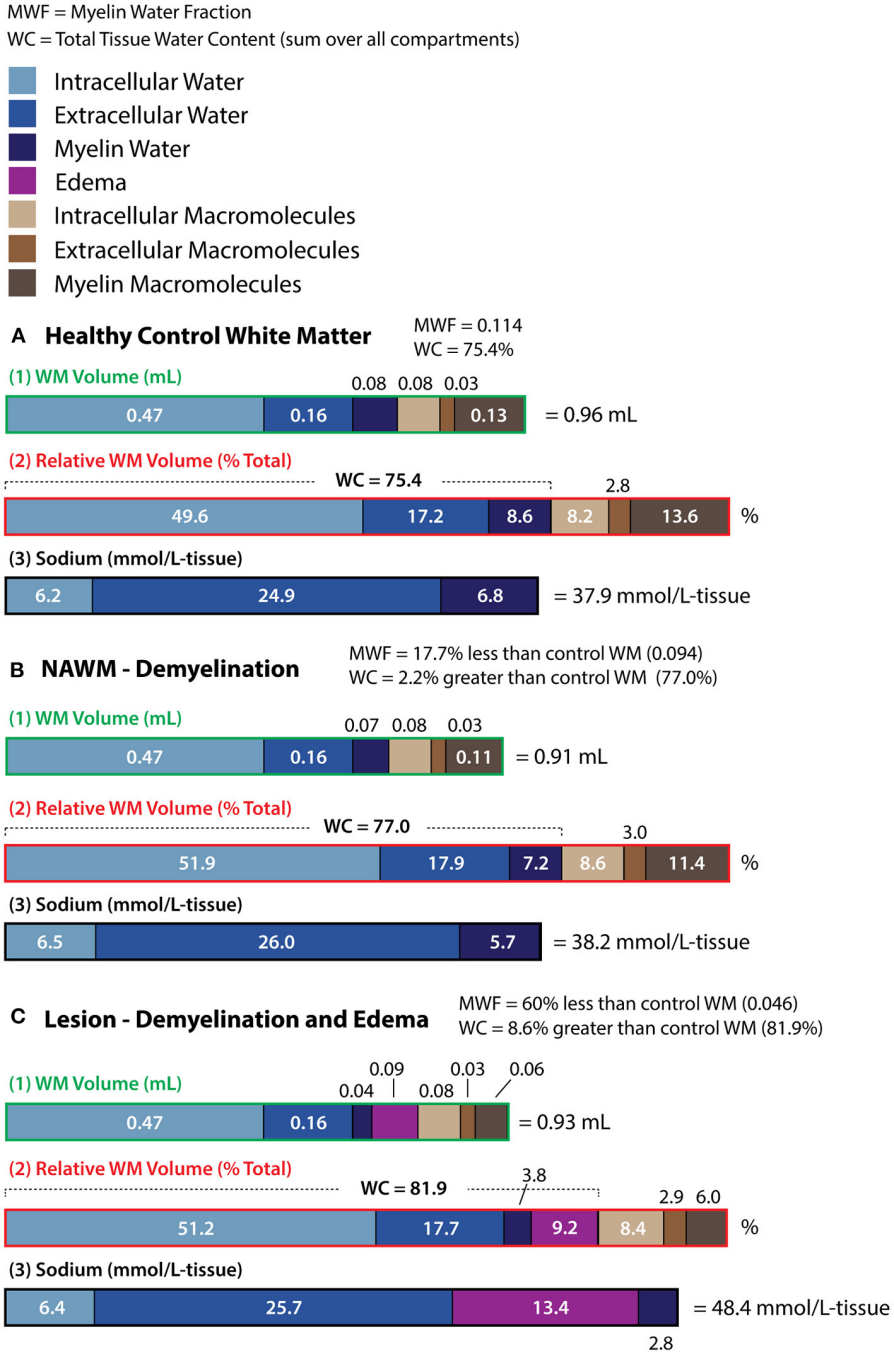
Compartmental white matter (WM) volumes referenced to 1 gram of healthy tissue (label “1” and green), relative WM volumes as a percentage of total tissue volume (label “2” and red), and sodium concentrations (label “3” and black) are given for models of **(A)** healthy control white matter, **(B)** NAWM, and **(C)** lesions. Compartmental white matter (WM) volumes are based on myelin water fraction (MWF) for **(A)** healthy control white matter, and both MWF and water content (WC) difference from healthy control WM for **(B)** NAWM and **(C)** lesions. White matter volumes were calculated according to the T_2_ study of MS in reference (29), and the volumes for both **(A)** and **(B)** are also presented in (29). For the lesion case **(C)**, both demyelination and edema are required to model MWF and WC difference values derived from (29). Note that for healthy control WM **(A)**, the myelin sodium concentration is as great as that of intracellular sodium. Demyelination reduces myelin sodium, but when the loss of myelin macromolecules is associated with tissue atrophy [4.3% for NAWM in **(B)**], the relative fraction of space occupied by both intra- and extracellular sodium is increased (even if the absolute volumes of these spaces remain unchanged), slightly increasing tissue sodium concentration (in mmol/L tissue). For the lesion model **(C)**, considerably greater TSC is primarily driven by edema.

Tissue volume fractions of 9% (myelin water), 17% (extracellular water) and 50% (intracellular water) can be calculated using the framework presented by Laule et al. in the study of myelin water fraction (MWF) with T_2_ relaxometry (29) and the extracellular volume fraction measurements of (30, 31). Please see the associated Supplementary Section 1 for a description of this calculation. In Figure 7A1, volumes are listed for 1 gram of healthy control white matter, while the percentage of total tissue volume occupied by each component is shown in Figure 7A2. Intracellular water occupies only 50% of white matter volume, and thus intracellular sodium contributes only 0.5 L ^*^ 12.5 mM = 6.2 mmol to 1 L of tissue. This is listed as 6.2 mmol/L-tissue in Figure 7A3. It should be noted that although myelin water occupies a much smaller fraction of tissue space than intracellular water, it contributes a larger 6.8 mmol to 1 L of tissue than intracellular water (given its higher average concentration of 78.75 mM). The calculated total sodium contribution to 1 L of tissue is 37.9 mmol. This value is similar to the 40 ± 1 mmol/L-tissue experimentally measured here. For MWF = 0.2, as may be expected in the PLIC (32), the same calculation yields a “myelin sodium” of 10.9 mmol/L-tissue. This is almost 1/3 of the total calculated TSC = 40.5 mmol/L-tissue, and highlights the relevance of the myelin water compartment to the ultimate image contrast. Note that the software used in these calculations has been made available on-line (https://github.com/rstobbe/SodiumMyelinWater). Measurement or calculation of sodium within the myelin water space has not, to our knowledge, previously been reported in the literature.

To explore the underlying source(s) of image contrast in the three sequences, it is necessary to estimate the sequence relaxation weighting in each compartment. This estimation is accomplished with the spin-3/2 simulation software used and tested in each of the papers where the NaDW, NaPACMAN and NaSIRFLA sequences were introduced (17, 18, 20). This spin-3/2 simulation software has been made available on-line (https://github.com/rstobbe/TripleS). Please see the associated Supplementary Section 2 for a description of the relaxation models used for simulation. Relaxation weighting per compartment is given in Table 2 for each sequence. It is interesting to note that the NaPACMAN sequence with its large 110° flip-angle and long RF pulse duration τ_RF_ = 4.0 ms yields effectively no signal from the myelin water space for this model. In contrast, “partial inversion” (due to residual quadrupole splitting) and T_1_ recovery from a less negative M_z_ (18) results in elevated signal from the myelin water space model for NaSIRFLA. Intra- and extracellular relaxation weightings are the same and were derived from total tissue relaxation measurements. The relative signal contribution from each sodium MRI compartment can be modeled by combining the sequence relaxation weighting of Table 2 with the healthy control white matter sodium contribution from Figure 7A3. Relative signal contributions are given in Table 3 in proportion to the relative healthy control signal contribution from intracellular space for each sequence.

**Table 2:** Relaxation weighting (RW) of each sequence (i.e., signal relative to 1) for four different ^23^Na environments determined according to Supplementary Section 4.4.2.

	**Intra RW**	**Extra RW**	**Myelin RW**	**Edema RW**
NaDW	0.95	0.95	0.85	0.96
NaPACMAN	0.35	0.35	0.01	0.40
NaSIRFLA	0.45	0.45	0.61	0.10

**Table 3:** Relative sodium signal contributions (arbitrary units) from each compartment for each ^23^Na sequence are calculated from the modeled sodium contributions to tissue of Figure 7 (in mmol/L-tissue) and the relaxation weightings of Table 2, and are given in proportion to the relative healthy control intracellular signal contribution for each sequence (rounded to 2 decimal places).

	**Intra**	**Extra**	**Myelin**	**Edema**	**Total**	**% Difference (vs. control)**
NaDW (Control)	1.00	4.01	0.98	-	5.99	-
NaDW (NAWM)	1.05	4.20	0.82	-	6.06	1.2%
NaDW (Lesion)	1.03	4.14	0.43	2.17	7.78	30%
NaPACMAN (Control)	1.00	4.01	0.03	-	5.05	-
NaPACMAN (NAWM)	1.05	4.20	0.03	-	5.27	4.4%
NaPACMAN (Lesion)	1.03	4.14	0.01	2.46	7.65	52%
NaSIRFLA (Control)	1.00	4.01	1.48	-	6.49	-
NaSIRFLA (NAWM)	1.05	4.20	1.24	-	6.49	0%
NaSIRFLA (Lesion)	1.03	4.14	0.65	0.48	6.30	−3%

*The total relative signal models the relative ^23^Na MRI intensity for each sequence and the relative image intensity difference between Control WM, NAWM and lesions*.

NAWM volumes for the demyelination model of (29) are described in the associated Supplementary Section 1. Within this model the volumes of intra- and extracellular space remain unchanged (Figure 7B1), but due to the loss of myelin water and macromolecules (and associated tissue atrophy) the relative volume fractions of intra- and extracellular space are increased (Figure 7B2). Thus, both intra- and extracellular space contribute greater sodium to 1L of tissue (Figure 7B3) than they do in the case of healthy WM (Figure 7A3). However, this is offset by the reduced sodium contribution from the smaller myelin water tissue volume fraction. The result is a very small TSC increase of 0.8%. When the relaxation weighting of NaDW is taken into account, the signal increase is 1.2% (Table 3). However, the relaxation weighting of NaPACMAN yields 4.4% greater NAWM signal. This is because NaPACMAN reflects only the greater sodium contribution from the larger intra- and extracellular tissue volume fraction, and does not reflect the reduced myelin sodium contribution. For NaSIRFLA, signal from myelin sodium is enhanced compared to intra- and extracellular space (Table 2), and thus the loss of myelin sodium has greater impact yielding no NaSIRFLA signal change. Trends for 5% (NaDW) and 8% (NaPACMAN) NAWM increase over control WM that did not survive FDR correction are visible in Figures 3A,B. These trends reflect the simulation results. Zero difference for NaSIRFLA also reflects the experimental result shown in Figure 3C.

A white matter lesion model which includes both demyelination and edema can explain the myelin water fraction and water content measurements in (29). This model is described in the associated Supplementary Section 1 and is shown in Figure 7C. Calculated image intensities are shown for this lesion model in Table 3. The much greater sodium signal difference of lesions vs. control WM for NaPACMAN (52%) than NaDW (30%) reflects the experimentally measured relative results of 75 and 35%, respectively, in all MS patients, as does the calculated lower signal in lesions for NaSIRFLA (−3%) relative to an experimental trend of −6% (Figure 3). For NaSIRFLA, lower signal is the result of strong relaxation weighting in edema. Even greater TSC and image intensity increases (particularly for NaPACMAN) would be associated with axon degeneration, which is not included in this model. Note that lesions within this study were not separated into different types (e.g., acute vs. chronic).

### PASAT—NAWM Sodium MRI Correlation

The PASAT test provides a measure of attentional processing and is widely used in multiple sclerosis (33). In Figure 6C, reduced processing rates are associated with lower NaSIRFLA signal in the NAWM of the PLIC and across the average of the three WM regions considered. One might assume a lower PASAT score to be associated with demyelination, as demyelination results in over-expressed voltage-gated sodium channels along demyelinated axons and increased energy demand for action potential transmission (14). The NAWM demyelination model of Figure 7 and the sequence simulation of Table 3 point to greater NaDW (TSC) and particularly NaPACMAN signal with greater demyelination, and thus if lower PASAT score is associated with demyelination, one would expect greater NaDW and NaPACMAN signal with lower PASAT score. However, no significant negative correlations between PASAT and NaDW or NaPACMAN were measured (Figures 6A,B). Note that the trend observed in Figure 6A for NaDW in the ALIC is a *positive* trend (opposite that expected). Greater intracellular sodium concentration might be expected from the over-expression of voltage-gated sodium channels along demyelinated axons if the Na+/K+ ATPase pumps cannot “keep up,” and thus one might expect an association between greater intracellular sodium concentration and lower PASAT score. If NaSIRFLA is intracellular weighted (as has previously been suggested), this should be reflected as a negative correlation between NaSIRFLA and PASAT. However, the correlation of Figure 6C is positive. This suggests that lower PASAT score might be associated with lower intracellular sodium concentration. An alternative hypothesis for the positive correlation between NaSIRFLA and PASAT concerns the sodium concentration of extracellular space. Mild hyponatremia has been associated with cognitive impairment (34), and if lower extracellular sodium concentration was associated with reduced PASAT score in the MS patients studied here, this would yield a positive correlation with NaSIRFLA. In this case, one would also expect a positive correlation with PASAT for NaDW and NaPACMAN, but for these sequences the opposing (signal increasing) effects of demyelination may mask this association.

A previous study measured significant negative PASAT correlation with TSC across all NAWM of MS patients as identified by T_1_ masking (3), i.e., greater TSC associated with reduced attentional processing. This other study also measured negative 9HPT (9 hole peg test) and MSFC (multiple sclerosis functional composite) correlation with NAWM TSC. In addition, significantly greater NAWM TSC has also been measured in cognitively impaired MS patients when compared to either cognitively preserved MS patients or healthy controls (8). While other MS studies have also measured negative correlation between clinical/cognitive scores and cortical gray matter TSC (3, 8, 11), cortical gray matter was not assessed in this study. Given the low resolution and substantial PSF smearing in ^23^Na MRI images, the proximity of cortical gray matter to CSF may lead to atrophy biases.

### Conclusion

Advanced sodium MRI imaging with relaxation-weighted signal dependence beyond tissue sodium concentration may help elucidate the sources of sodium concentration change in Multiple Sclerosis and provide additional insight into this pathology as well as in other brain disorders.

## Data Availability Statement

The raw data supporting the conclusions of this article will be made available by the authors, without undue reservation.

## Ethics Statement

The studies involving human participants were reviewed and approved by Research Ethics Office (REO) University of Alberta. The patients/participants provided their written informed consent to participate in this study.

## Author Contributions

RS study design, image acquisition and analysis, data analysis, and writing. AB data analysis and writing. PS patient recruitment and analysis. DE image acquisition and analysis. DV data analysis. CB study design, data analysis, and writing. All authors contributed to the article and approved the submitted version.

## Conflict of Interest

The authors declare that the research was conducted in the absence of any commercial or financial relationships that could be construed as a potential conflict of interest. The Handling Editor declared a past co-authorship with one of two authors, RS and CB.
