# Supplementary material for: Sodium Intensity Changes Differ Between Relaxation- and Density-Weighted MRI in Multiple Sclerosis
Source: Front Neurol. 2021 Jul 14;12:693447. doi: 10.3389/fneur.2021.693447 (PMC8323606; doi:10.3389/fneur.2021.693447)
Supplement: Supplementary file 1 [file Presentation_1.pdf]

1. White Matter Volume Fraction Calculation

Healthy Control White Matter

White matter tissue volume fractions were calculated for healthy tissue using the framework presented in the study of myelin water fraction (MWF) with T_2 relaxometry by Laule et. al. (Laule et al. 2004). Using values of myelin water content = 0.369 (g H_2O)/(g myelin) and non-myelin water content = 0.82 (g H_2O)/(g non-myelin), Laule et. al. calculated water and dry macromolecule fractions of tissue by weight for a given MWF. These weight fractions (in grams per gram of tissue) are given in Figure 6a of reference (Laule et al. 2004) for healthy control white matter with MWF = 0.114. Here myelin water accounts for 0.082 g, while non-myelin water accounts for 0.638 g. Myelin and non-myelin volumes can then be calculated for 1.0 gram of healthy control white matter using the density values of 1.08 for dry myelin and 1.33 for dry non-myelin (also from (Laule et al. 2004)). These tissue volumes are given in **Figure 7A1**, where non-myelin volume is the combination of both intra- and extracellular space (the splitting of which is described below). Given that the density of water is 1.0, the volumes of myelin and non-myelin water are 0.082 mL and 0.638 mL respectively, while the total tissue volume is 0.955 mL (also listed in Figure 6a of reference (Laule et al. 2004)). Thus, myelin water accounts for 9% of healthy control white matter volume (**Figure 7A2**). Non-myelin water (78% of the tissue volume) was then split into intra- and extracellular components such that an extracellular volume fraction (ECVF) of 20% was achieved (Sykova and Nicholson 2008; Nicholson and Hrabetova 2017), where this 20% includes both the extracellular water (17%) and the dry macromolecules (3%) in the ratio of 86% water and 14% dry macromolecules for non-myelin space (volume ratios calculated from the non-myelin water content and molecule densities given above). Subtracting the extracellular volume fraction from the non-myelin water volume fraction yields an intracellular volume fraction of 58%. Splitting this into water and dry macromolecule components using the same non-myelin volume ratios yields an intracellular water fraction of 50%. Summing all of the water spaces yields a total water fraction of 75%. (**Figure 7A2**).

NAWM with Demyelination

Using multi-exponential T_2 ‘myelin water imaging’, Laule et. al. experimentally measured 16% lower MWF and 2.2% greater tissue water content in NAWM compared to controls, and suggested

a demyelination model (Figure 6c in reference (Laule et al. 2004) and **Figure 7B**) explained this result better than either edema (Figure 6b in reference (Laule et al. 2004)) or cellular infiltrates (Figure 6d in reference (Laule et al. 2004)). However, a simple demyelination model can not simultaneously describe 16% lower MWF and 2.2% greater tissue water content, and thus Laule's model included a 17.7% lower MWF = 0.094, through which the 2.2% tissue water increase was attained. In this case, the loss of myelin yields a tissue volume of only 0.914 mL, describing tissue atrophy of 4.3% (also given in Figure 6c of reference (Laule et al. 2004)), a value reflecting previous study (Ge et al. 2001). As a result, the relative WM volume fraction associated with intra- and extracellular space is increased (**Figure 7B2**), even if the volumes of these spaces themselves remain the same (**Figure 7B1**).

Lesions with Demyelination and Edema

A model which includes both demyelination and edema explains both the lower MWF and greater water content in lesions. In **Figure 7C**, lesions are modelled using values extrapolated from (Laule et al. 2004). Note that while a mean lesion MWF = 0.046 was experimentally measured in (Laule et al. 2004), mean healthy control white matter does not represent the same directly comparable tissue. The lesion water increase of 8.6% (relative to control white matter) is derived from the measured 6.3% increase over contralateral NAWM, and the average 2.2% water increase in NAWM of specific structures compared to control white matter (i.e 1.086 = 1.063 * 1.022). Nevertheless, these values provide initial estimates for a model which includes both demyelination and edema to explain both lower MWF and greater water content in lesions.

2. ^{23}Na Relaxation Models Used to Estimate Sequence Weighting

The same (total tissue based) model is used for both intra- and extracellular space, and is derived from the biexponential T_2 fitting described in (Stobbe and Beaulieu 2016) and the T_1 fitting described in (Stobbe and Beaulieu 2006), yielding spectral density parameters of $J_0 = 0.392$, $J_1 = 0.025$, and $J_2 = 0.013$ (corresponding to $T_{2fast} = 2.4$ ms; $T_{2slow} = 20$ ms; $T_{1(mono)} \sim 36$ ms, where $T_{1(mono)}$ is a monoexponential approximation of T_1). Although a subcutaneously implanted gliosarcoma in rats (Winter and Bansal 2001) yielded (^{23}Na shift reagent enabled) intracellular relaxation of $T_{2fast} = 2$ ms, $T_1 = 24$ ms and extracellular relaxation of $T_{2fast} = 3.4$ ms, $T_1 = 46$ ms, this tumour model does not necessarily describe healthy brain tissue. Current opinion suggests that these two environments of intra- and extra-cellular space may not possess substantially different sodium relaxation (Burstein and Springer 2019). For this reason the same (total tissue based) model is used for both intra- and extracellular space. For the myelin water space, zero-mean Gaussian distributed residual quadrupole splitting with standard deviation of 625 Hz is added to the previous model, a data fitting estimate from reference (Stobbe and Beaulieu 2016). Finally a model for edema was also derived from relaxation measurements of blood plasma (Perman et al. 1986). Spectral density parameters of edema were taken as $J_0 = 0.074$, $J_1 = 0.0094$, $J_0 = 0.0094$, for $T_{2fast} = 12$ ms, and a $T_1 = 53$ ms similar to saline at 4.7T. Note that for each environment, ^{23}Na relaxation parameters remain to be directly measured. This may require new regression methods, such as described in (Kordzadeh et al. 2020). Nevertheless, 'best estimate' models are used to see if they might help explain experimental image contrast.

References

Laule, C., I. M. Vavasour, G. R. W. Moore, J. Oger, D. K. B. Li, D. W. Paty, and A. L. MacKay. 2004. 'Water content and myelin water fraction in multiple sclerosis - A T-2 relaxation study', *Journal of Neurology*, 251: 284-93.

Nicholson, C., and S. Hrabetova. 2017. 'Brain Extracellular Space: The Final Frontier of Neuroscience', *Biophysical Journal*, 113: 2133-42.

Perman, W. H., P. A. Turski, L. W. Houston, G. H. Glover, and C. E. Hayes. 1986. 'Methodology of Invivo Human Sodium Mr Imaging at 1.5 T', *Radiology*, 160: 811-20.

Stobbe, R. W., and C. Beaulieu. 2016. 'Residual quadrupole interaction in brain and its effect on quantitative sodium imaging', *Nmr in Biomedicine*, 29: 119-28.

Stobbe, R.W., and C. Beaulieu. 2006. 'Sodium Relaxometry: Towards the Characterization of the Sodium NMR Environment in the Human Brain Using a Novel Relaxometry Technique', *Proceedings of the 14th Annual Meeting of ISMRM, Seattle, Washington, USA*: 3104.

Sykova, E., and C. Nicholson. 2008. 'Diffusion in brain extracellular space', *Physiological Reviews*, 88: 1277-340.
